# *Lactobacillus fermentum* ZC529 Protects Intestinal Epithelial Barrier Integrity by Activating the Keap1-Nrf2 Signaling Pathway and Inhibiting the NF-κB Signaling Pathway

**DOI:** 10.3390/antiox14060732

**Published:** 2025-06-14

**Authors:** Zian Yuan, Lang Huang, Zhenguo Hu, Junhao Deng, Yehui Duan, Qian Jiang, Bi’e Tan, Xiaokang Ma, Chen Zhang, Xiongzhuo Tang

**Affiliations:** 1 Hunan Provincial Key Laboratory for the Products Quality Regulation of Livestock and Poultry, College of Animal Science and Technology, Hunan Agricultural University, Changsha 410128, China; 1287735078@stu.hunau.edu.cn (Z.Y.); huanglang@stu.hunau.edu.cn (L.H.); dengjunhao@stu.hunau.edu.cn (J.D.); jiangqian@hunau.edu.cn (Q.J.); bietan@hunau.edu.cn (B.T.); maxiaokang@hunau.edu.cn (X.M.); 2 Yuelushan Laboratory, Changsha 410128, China; 3 Faculty of Agriculture, Kagoshima University, Korimoto, Kagoshima 8900065, Japan; 4 Institute of Subtropical Agriculture, Chinese Academy of Sciences, Changsha 410125, China; huzhenguo22@mails.ucas.ac.cn (Z.H.); duanyehui@isa.ac.cn (Y.D.); 5 Institute of Yunnan Circular Agricultural Industry, Pu’er 665000, China; 6 Anhui Provincial Key Laboratory of Livestock and Poultry Product Safety, Institute of Animal Husbandry and Veterinary Medicine, Anhui Academy of Agricultural Sciences, Hefei 230011, China

**Keywords:** *Lactobacillus fermentum*, antioxidant, anti-inflammation, Nrf2, NF-κB

## Abstract

The probiotic bacteria *Lactobacillus fermentum* ZC529 (*L.f* ZC529) has been identified from the colon of the Diannan small-ear (DSE) pig, but its intestinal protective function still lacks investigation. Here, we established a dextran sodium sulfate (DSS)-induced intestinal oxidative stress model in both *Drosophila* and porcine small intestinal epithelial (IPEC-J2) cell lines to explore the anti-oxidative and anti-inflammatory effects of *L.f* ZC529. The data showed that the intestinal colonization of *L.f* ZC529 counteracted DSS-induced intestinal oxidative stress and excessive reactive oxygen species (ROS) generation by activation of the CncC pathway, a homology of the nuclear factor erythroid 2-related factor 2 (Nrf2) in mammalian systems. Moreover, *L.f* ZC529 supplementation prevented flies from DSS-induced intestinal barrier damage, inflammation, abnormal excretory function, and shortened lifespan. Finally, *L.f* ZC529 also attenuated DSS-induced intestinal injury in the IPEC-J2 cell line by activating the Keap1-Nrf2 signaling and inhibiting the NF-κB signaling pathways. Together, this study unraveled the profound intestinal protective function of *L.f* ZC529 and provides its potential application as a new antioxidant in improving animal intestinal health as well as in developing a new probiotic in the food industry.

## 1. Introduction

The maintenance of intestinal steady state is dependent on the balance between the intestinal immune system and the gut microbiota. Disrupted immune activities, including excessive oxidative stress, are often linked to intestinal inflammatory diseases such as inflammatory bowel disease in humans and diarrhea in post-weaning piglets [1]. Intestinal epithelial cells act as an immune layer to defend against external environmental challenges and are responsible for preserving the integrity of the intestinal barrier [2]. The Keap1-Nrf2 pathway is the major sensor of cellular oxidative stresses, and the activation of this pathway can enhance antioxidant capacity by the upregulation of cytoprotective gene expression, thus protecting intestinal epithelium from oxidative stress-induced injuries [3]. Nrf2 is a key transcription factor that regulates the expression of various detoxifying and antioxidant enzymes, including glutathione S-transferases (GSTs), heme oxygenase-1 (HO-1), and glutathione synthetase (GCS) [4]. The activity of the Keap1-Nrf2 pathway plays a key function in maintaining intracellular redox homeostasis and mitigating oxidative stress-induced damages in cells.

In addition to the Keap1-Nrf2 pathway, the TLR4-NF-κB signaling axis also plays a crucial role in regulating immune responses and inflammatory processes in the gut upon various stimuli [5]. The TLR4-NF-κB pathway is involved in the production of pro-inflammatory cytokines such as interleukin-1 beta (IL-1β), interleukin-6 (IL-6), and tumor necrosis factor alpha (TNF-α), which contribute to the development and progression of inflammation [6]. The inhibition of the TLR4-NF-κB axis has been shown to attenuate the inflammatory response and protect against intestinal injury [7]. Therefore, both the Keap1-Nrf2 and NF-κB pathways are crucial for preserving intestinal barrier function and preventing excessive inflammatory processes [8,9].

The Diannan small-ear (DSE) pig, one of the Chinese indigenous pig breeds, exhibits high stress and immune resistance as well as high crude fiber digestibility in the gut, but the underlying mechanisms are not fully understood [10]. We have previously isolated a specific gram-positive bacterial strain, *Lactobacillus fermentum mucilaginosus* (*L.f* ZC529), from the colonic chyme of the DSE pig and characterized its biomedical and probiotic activities in vitro [11,12]. *L.f* ZC529 displays a high free-radical scavenging capacity against *2,2-diphenyl-1-picrylhydrazyl* (DPPH), superoxide, and hydroxyl radicals [13]. However, the in vivo anti-oxidative activity of *L.f* ZC529 is still not known.

*Drosophila melanogaster* has been considered as an excellent genetic model to study genes’ expression and their biological processes in the gut, due to its conserved intestinal anatomy and function, as well as numerous well-developed genetic tools [14,15,16]. Oxidative damages induced by a variety of environmental stimuli, including DSS, paraquat, sodium dodecyl sulfate (SDS), and pathogenic bacteria often cause deleterious injury in the *Drosophila* gut epithelium, leading to inflammation and shortened lifespan [17]. A subset of immune and signaling pathways such as Keap1-Nrf2 and Myd88-NF-κB pathways are involved in intestinal epithelial repair to maintain gut homeostasis [18]. In addition, the porcine intestinal epithelial (IPEC-J2) cell line, which was originally derived from jejunal epithelia, has been utilized as an ideal in vitro model to investigate how nutritional intervention or environmental stress affects intestinal epithelial function [19,20]. Therefore, *Drosophila* and IPEC-J2 cells were utilized to examine the anti-oxidative and anti-inflammatory function of *L.f* ZC529 in response to DSS-induced intestinal oxidative damage.

In this study, we characterized the anti-oxidative and anti-inflammatory effects of *L.f* ZC529 in response to DSS treatment in both *Drosophila* and IPEC-J2 cells. The pre-colonization or culture of *L.f* ZC529 alleviated DSS-induced oxidative damage and inflammation in the intestinal epithelial cells by the activation of the Keap1-Nrf2 and NF-κB signaling pathways. Our study expanded the understanding of the mechanism of stress resistance in the DSE pig and also highlighted the potential application of *L.f* ZC529 as a new antioxidant to protect the intestine from oxidative stress-induced injury.

## 2. Methods and Materials

### 2.1. Culture and Preparation of Lactobacillus fermentum ZC529

The *Lactobacillus fermentum* ZC529 strain was preserved at −80 °C in 20% (*v*/*v*) glycerol as described by Zhang et al. [12]. For the experimental culture, *L.f* ZC529 was streak-plated on de Man–Rogosa–Sharpe (MRS) agar and incubated anaerobically (GasPak EZ, BD, Franklin Lakes, NJ, USA) at 37 °C for 24 h. A single colony was transferred to 50 mL of MRS broth and cultured at 37 °C in a shaker with 150 rpm until mid-log phase (OD_600_ ≈ 1.2, approximately 1 × 10^9^ CFU /mL). The cells were harvested by centrifugation (4000× *g*, 10 min, 4 °C), washed three times with sterile phosphate-buffered saline (PBS, pH 7.4), and resuspended in antibiotic-free DMEM/F-12 medium. Immediately before use, viable counts were confirmed by serial dilution and plate enumeration, and the suspension was adjusted to the desired multiplicity of infection (MOI; standard condition = 100 bacteria: 1 cell).

### 2.2. Fly Husbandry and Survival Assay

The *gstd1*-GFP transgenic reporter strain was provided by the Animal Nutrition Genome and Germplasm Innovation Center, Hunan Agricultural University (Changsha, China). Flies were reared at 25 degrees in 60% relative humidity on a 12/12 h light/dark cycle on instant medium.

For the intestinal pre-colonization of *L.f* ZC529 in flies. Firstly, 3–5-day-old female flies were starved for 2 h in empty vials at 29 °C in an incubator and then briefly anesthetized to allow transfer to new vials. Next, flies were fed with 300 µL of feeding solution (1:1 mixture of *L.f.* ZC529 and 5% sucrose) for four days before 5% (*w*/*v*) dextran sodium sulfate (DSS, MW 36–50 kDa; MP Biomedicals, cat. #160110, Solon, OH, USA) treatment. Flies were fed once with fresh feeding solution every day during the colonization period.

The survival assay was performed in conventional rearing conditions, with a mixture of 3–5-day-old flies, 25 females and 5 males, in each vial. Briefly, at least three biological replicates of each group (control group, 5%DSS group, *L.f* group, *L.f*+5%DSS group) were placed in an empty vial containing a piece of disk filter paper. The flies were starved in empty vials at 29 degrees for 2 h before feeding with 300 μL of feeding medium. A total of 5% of sucrose solution was used as control, 5% DSS diluted in 5% sucrose was used as the chemical treatment group, and *L.f* culture concentration at OD_600_ ≈ 1.2 was utilized as the *L.f* group. All flies were transferred to new vials with fresh feeding medium every day. The number of dead flies in each vial was recorded daily to calculate the survival rate. Both male and female flies were used, and the mortality data were not sex-specific. Midguts were dissected from flies in each group (*n* = 6–8 per group) and stained with DAPI to assess the *gstd1*-GFP fluorescence intensity.

### 2.3. Smurf Assay

The detailed “Smurf” assay was performed as described by Livingston et al. [21]. Briefly, 3–5-day-old flies, 20 flies of each group, were starved at 29 degrees for 2 h before feeding with 300 μL of medium containing 2.5% of blue dye (FD&C Blue No. 1; Brilliant Blue FCF; Sigma-Aldrich, St. Louis, MO, USA; cat. #B4529). All flies were transferred to a new culture tube daily. Flies were anesthetized and examined under a stereomicroscope. A fly was counted as Smurf-positive only when blue dye had leaked beyond the gut lumen and diffusely stained both the abdomen and thorax cuticle, whereas dye restricted to the digestive tract was considered Smurf-negative; two blinded observers scored each vial independently (concordance > 95%, discrepancies resolved by a third scorer), and the percentage of Smurf-positive flies was calculated from at least three biological replicates per treatment.

### 2.4. Excretion Assay

To assess the excretory capacity of the *Drosophila* gut, we used the *Drosophila* excretory method described in [22]. Firstly, two circular chromatography papers of the same size as the culture tube were clamped to the bottom of the tube, followed by 200 µL of 2% Brilliant Blue solution on the surface of the culture medium to ensure complete absorption of the solution. The paper was then cut into 3.7 cm × 5.8 cm pieces and placed next to the wall of the food tube to ensure that it did not come into contact with the Brilliant-Blue-treated food. After one hour of starvation, the flies were allowed to feed on the medium for 24 h. At the end of the incubation period, the excreta-covered papers were removed and imaged immediately with a Leica M205 FA stereomicroscope equipped with a DFC450 C camera at 1.25 × magnification and identical exposure settings; TIFF files were imported into FIJI/ImageJ, converted to 8-bit, and a color threshold (Hue 160–200, Saturation 40–255, Brightness 0–255) was applied to isolate Brilliant Blue deposits; the “Analyze Particles” function (size ≥ 0.02 mm^2^, circularity 0.20–1.00) automatically counted and measured each spot, yielding the total spot number and cumulative stained area per paper, which were then normalized to the number of flies in the vial and expressed as “spots fly^−1^ 24 h^−1^” or “stained area (mm^2^) fly^−1^ 24 h^−1^”; background threshold and particle parameters were calibrated on control papers lacking excreta and kept constantly for all analyses. The quantification was performed blindly on three independent biological replicates per treatment.

### 2.5. Cell Pretreatment

IPEC-J2 cells (Cell Bank of the Chinese Academy of Sciences, Shanghai; Cellosaurus accession CVCL_2246) were routinely maintained in Dulbecco’s Modified Eagle Medium/Nutrient Mixture F-12 (DMEM/F-12; Gibco #11885084) supplemented with 10% (*v*/*v*) heat-inactivated fetal bovine serum (FBS; Gibco #A3160901) and 1% antibiotic cocktail (10 kU mL^−1^ penicillin, 10 mg mL^−1^ streptomycin, 25 µg mL^−1^ amphotericin B; Gibco #15070063, #15290026). Cells were incubated at 37 °C with a continuous supply of 5% CO_2_. The antibiotics served only for routine maintenance and were completely removed 12 h before any probiotic treatment to preclude interference with bacterial viability.

For co-culture, sub-confluent monolayers (70–80%) were washed once with warm phosphate-buffered saline (PBS) and incubated with *L.f* ZC529 (prepared as in Section 2.1) at a multiplicity of infection (MOI) of 100 bacteria: 1 cell in antibiotic-free DMEM/F-12 medium for 3 h at 37 °C, with 5% CO_2_. Unattached bacteria were removed by gentle PBS rinsing, after which the cells were challenged with 5% DSS for 12 h unless otherwise indicated. Vehicle-treated controls underwent identical medium changes without bacteria or DSS exposure. All experiments were performed in four independent biological replicates.

### 2.6. Measurement of Intracellular Reactive Oxygen Species (ROS) Levels in Cells

Fluorescence microscopy imaging in conjunction with image analysis software were employed to ascertain the quantitative level of reactive oxygen species within IPEC-J2 cells. IPEC-J2 cells were cultured until they reached 80–90% confluency in Dulbecco’s Modified Eagle Medium (GIBCO, Grand Island, NY, USA, #11885084) containing 10% fetal bovine serum (GIBCO, Grand Island, NY, USA, #A3160901). The experimental group was treated with 5% dextran sodium sulfate (MP Biomedicals, Solon, Ohio, USA, #9011-18-1) to induce ROS production, while the control group was treated with DSS-free medium. The fluorescent labeling of ROS was incubated at 37 °C for 30 min with 2′,7′-dichlorofluorescin diacetate (Biyuntian, China, #S0033S). Subsequently, fluorescence images of each sample were acquired using a fluorescence microscope Axio Scope 5(Zeiss, Oberkochen, Germany, #2020001895), and the same exposure conditions were maintained for all samples. The images were then processed using Image J (National Institutes of Health, Bethesda, MD, USA; https://imagej.net/ij/ accessed date 9 May 2025). Finally, the ROS levels of the different treatment groups were quantitatively compared and analyzed, and significant differences were determined.

### 2.7. Real-Time Quantitative PCR (RT-qPCR)

*Drosophila* intestines were carefully isolated and then immediately immersed in pre-cooled PBS to prevent degradation. The tissue lysis was performed using Trizol reagent (Invitrogen Corporation, Grand Island, NY, USA) followed by mechanical disruption by grinding. The RNA was then extracted from other cellular components using chloroform, precipitated with isopropanol, and washed with 70% ethanol. Subsequently, RNA was resuspended in DEPC-treated water, and DNase treatment was performed to eliminate DNA contamination. RT-qPCR was performed on a CFX96TM system (BIO-RAD, Hercules, CA, USA) using ChamQ Universal SYBR qPCR Master Mix (Vazyme, Nanjing, China, #Q711-02). The amplification program consisted of an initial denaturation at 95 °C for 30 s, followed by 40 cycles of 95 °C for 10 s and 60 °C for 30 s, and a melt-curve step from 65 °C to 95 °C (0.5 °C s^−1^) to confirm single-product specificity. The relative mRNA expression levels of the target genes were calculated using the 2^−ΔΔCT^ method, with *Rp49* gene as the endogenous control [23].

The IPEC-J2 cells were cultured in the 6-well plate at a concentration of 2 × 10^5^ cells/well. The total RNA of IPEC-J2 cells was extracted using Trizol (Invitrogen Corporation, Grand Island, NY, USA). Then, the total RNA was reverse-transcribed to cDNA according to the instructions of Takara Prime Script RT reagent Kit (Dalian, China). Next, the following qPCR processes were performed as described above. All primer pairs were adopted from published studies listed in Table 1 and Table 2.

### 2.8. Anti-Oxidative Activity Measurement in Flies

The guts from flies were dissected and homogenized in cold PBS, and then the supernatant were collected at 4 °C, 3000 r/min, with centrifugation for 10 min. Subsequently, the activities of SOD, CAT, GSH, MDA, NOX, and TAOC were measured in kits according to the instructions (Jiancheng, Nanjing, China, #A00132, #A00711, #A00621, #A00312, #A11611, #A01521).

### 2.9. Anti-Oxidative Activity Measurement in IPEC-J2 Cells

The IPEC-J2 cells were lysed using RIPA High Efficiency Cell Lysis Buffer (Beyotime, Shanghai, China), and the lysates were centrifuged to obtain the supernatants. The levels of oxidative stress-related markers, including SOD, MDA, TAOC, SOD2, and CAT, were quantified using commercially available assay kits according to the manufacturer’s instructions (Jiancheng Bioengineering Institute, Nanjing, China; #A00132, #A00341, #A01521, #A00121, #A00711).

### 2.10. Statistical Analyses

All experiments were repeated at least three times. All data are presented as means ± standard error of mean (SEM). The statistical significance was analyzed using GraphPad Prism version 8.0 (San Diego, CA, USA). The one-way ANOVA test was performed to analyze differences between groups. The lifespan assays were tested for significance with a log-rank test; *p* < 0.05 was considered statistically significant, * *p* < 0.05, ** *p* < 0.01, *** *p* <0.001, and **** *p* < 0.0001. Non-significance (NS) represents *p* > 0.05. Error bars represent SEM.

## 3. Results

### 3.1. L.f ZC529 Alleviates DSS-Induced Intestinal Oxidative Stress in Drosophila

**Figure 1:**
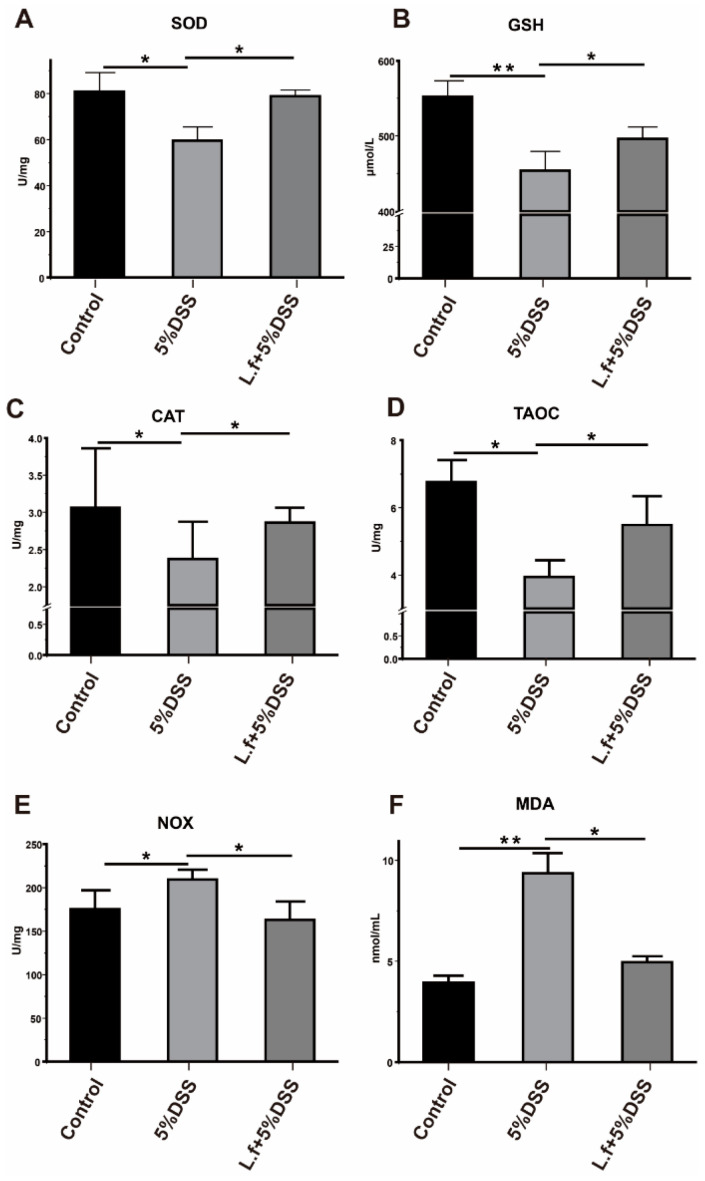
*L.f* ZC529 alleviates DSS-induced intestinal oxidative stress in *Drosophila*. (**A**–**F**) Measurement of the activity of antioxidant enzymes SOD (**A**), GSH (**B**), CAT (**C**), TAOC (**D**), NOX (**E**), and MDA (**F**) in the *Drosophila* gut. Asterisks denote statistically significant differences (* *p* < 0.05, ** *p* < 0.01). *p*-values were calculated by one-way ANOVA followed by Tukey’s post hoc test; Error bars denote mean ± SEM. *n* = 4 independent experiments.

To examine the anti-oxidative activity of *L.f* ZC529, we measured the activity of several antioxidant enzymes, including superoxide dismutase (SOD), glutathione (GSH), catalase (CAT), and total antioxidant capacity (TAOC) in the wild-type *Drosophila* gut upon 5% DSS treatment. As the positive control, the activities of SOD, GSH, CAT, and TAOC were significantly decreased after 5% DSS treatment (Figure 1A–D), while *L.f* ZC529 colonization restored the activity of these antioxidant enzymes in the gut (Figure 1B,D). In contrast, the activities of malondialdehyde (MDA) and NADPH oxidase (NOX), two major sources of cellular reactive oxygen species (ROS), were significantly increased in the gut in response to 5% DSS treatment, but *L.f* ZC529 colonization ameliorated the activity of these two enzymes in the gut (Figure 1E,F). These data suggest that *L.f* ZC529 alleviates DSS-induced oxidative stress in the *Drosophila* gut.

### 3.2. L.f ZC529 Mitigates ROS Generation by Activation of the CncC Pathway

**Figure 2:**
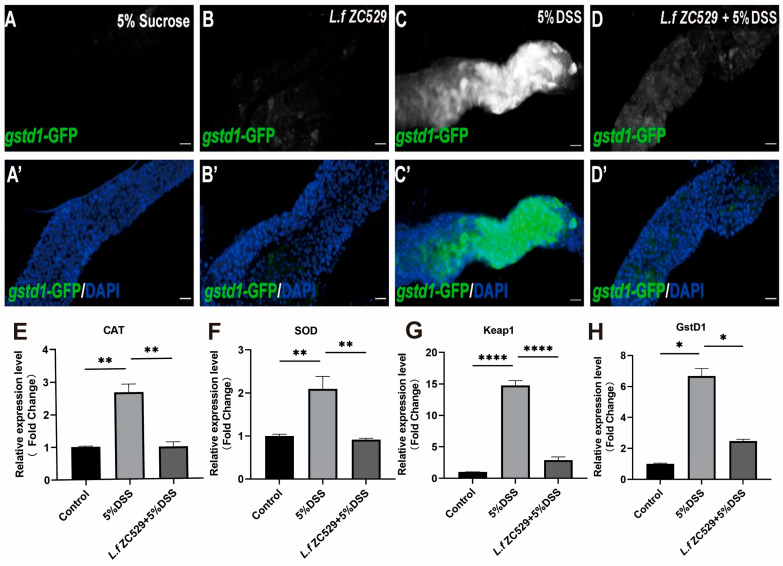
*L.f* ZC529 mitigates ROS generation by activation of the *CncC* pathway. (**A**–**D’**) The fluorescent intensity of the *gstd1*-GFP reporter line in the gut of control (**A**,**A’**), *L.f* ZC529 colonization (**B**,**B’**), 5%DSS treatment (**C**,**C’**), and 5%DSS+ *L.f* ZC529 groups (**D**,**D’**). (**E**–**H**) RT-qPCR measurement of antioxidant enzymes *CAT* (**E**), *SOD* (**F**), and *Keap1* (**G**) and its target gene *gstd1* expression in the gut of control and experimental groups. Asterisks denote statistically significant differences (* *p* < 0.05, ** *p* < 0.01, **** *p* < 0.0001). *p*-values were calculated by one-way ANOVA followed by Tukey’s post hoc test; error bars denote mean ± SEM. *n* = 4 independent experiments. Scale bars: 200 μm.

To investigate if *L.f* ZC529 is involved in the modulation of ROS levels upon DSS treatment, we examined the fluorescent signal of the *gstd1*-GFP transgenic flies, a reporter line reflecting the ROS level respondse to the *CncC* (also known as Nrf2 in mammalian system) pathway in the *Drosophila* gut. As expected, 5% DSS treatment induced a strong *gstd1*-GFP fluorescence in the gut when compared to control (Figure 2A–C’). While intestinal *L.f* ZC529 colonization alone slightly increased the *gstD1*-GFP signaling in the gut (Figure 2B,B’). Strikingly, flies pre-colonized with *L.f* ZC529 greatly reduced the high intensity of *gstd1*-GFP signal induced by 5%DSS treatment (Figure 2D,D’). The Nrf2-Keap1 signaling pathway protects against oxidative damage by promoting the gene expression of antioxidant enzymes to maintain intracellular redox homeostasis [29]. Therefore, we measured the expression of antioxidant genes CAT and SOD and the *CncC* pathway regulatory genes Keap1 and Gstd1 in the gut of *gstd1*-GFP transgenic flies. As shown in Figure 2E,H, 5% DSS treatment alone drastically increased CAT, SOD, Keap1, and Gstd1 expression in the gut. But *L.f* ZC529 pre-colonization decreased the expression of these antioxidative genes in the gut (Figure 2E,H). These results suggest that *L.f* ZC529 mitigates DSS-induced ROS levels by activating the *CncC* pathway in the *Drosophila* gut.

### 3.3. L.f ZC529 Prevents Flies from DSS-Induced Intestinal Epithelium Damage and Barrier Disruption

**Figure 3:**
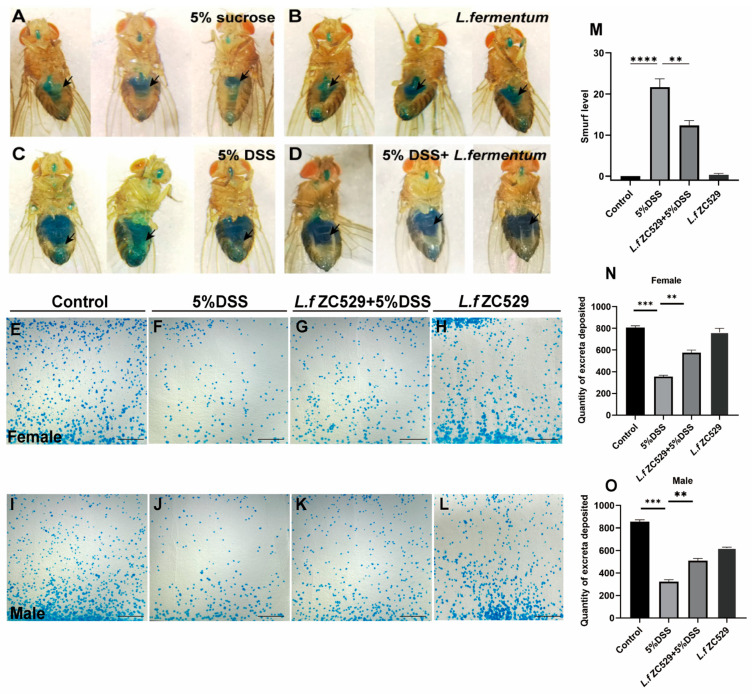
*L.f* ZC529 prevents flies from DSS-induced intestinal epithelial damage and barrier disruption. (**A**–**D**) Representative images of Smurf assays in the flies of control (**A**), *L.f* ZC529 colonization (**B**), 5%DSS treatment (**C**), and 5%DSS+ *L.f* ZC529 groups (**D**). (**M**) Quantification of the percentage of “Smurf” flies in control and experimental groups. (**E**–**H**) Representative images of excretion deposits in female control (**E**), 5%DSS treatment (**F**), *L.f* ZC529 colonization (**G**), and 5%DSS+ *L.f* ZC529 (**H**) groups and their quantification data (**N**). (**I**–**L**) Representative images of excretion deposits in male control (**I**), 5%DSS treatment (**J**), *L.f* ZC529 colonization (**K**), and 5%DSS+ *L.f* ZC529 (**L**) groups and their quantification data (**O**). Asterisks denote statistically significant differences (** *p* < 0.01, *** *p* < 0.001, **** *p* < 0.0001). *p*-values were calculated by one-way ANOVA followed by Tukey’s post hoc test; error bars denote mean ± SEM. *n* = 4 independent experiments. Scale bars: 700 μm.

To assess intestinal barrier integrity, we performed the “Smurf” assay [30]. In control flies, the blue food dye remained confined to the midgut (Figure 3A). While 5% DSS treatment flies exhibited a clear gut leakage phenotype as shown by the presence of blue dye in the whole abdomen (Figure 3C). Strikingly, feeding flies with *L.f* ZC529 for a week before 5% DSS treatment significantly alleviated the gut leakage phenotype (Figure 3B,D,M). In addition, we found that DSS-induced gut damage impaired excretory function both in females (Figure 3E–H) and males (Figure 3I–L), with less excretion deposits in the DSS-stimulated group when compared to control flies, but dietary supplementation of *L.f* ZC529 protected the excretory function of the gut upon DSS treatment (Figure 3N,O). These data suggest that pre-colonized *L.f* ZC529 may protect the gut from DSS-induced damage and contribute to the maintenance of gut epithelium integrity and normal excretory function.

### 3.4. L.f ZC529 Alleviates DSS-Induced Intestinal Inflammation and Restores the Lifespan of Drosophila

**Figure 4:**
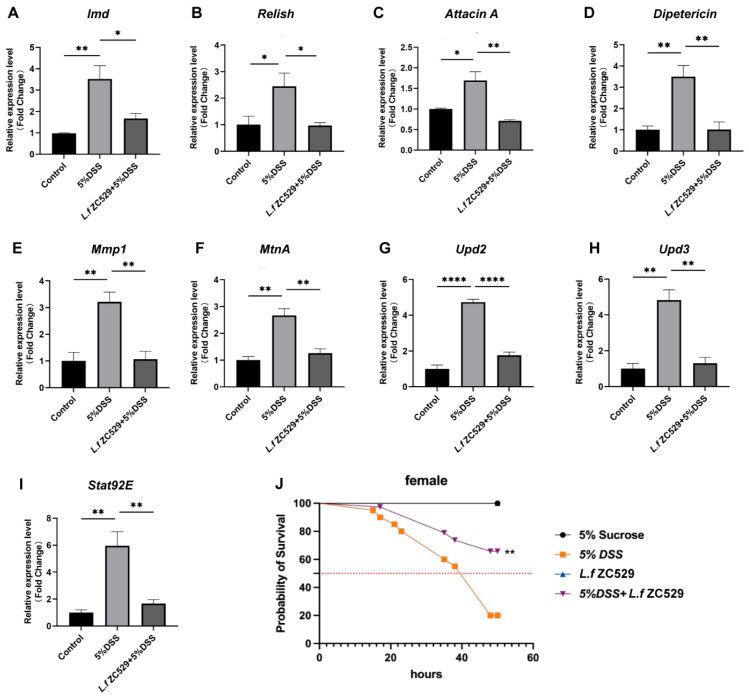
*L.f* ZC529 alleviates DSS-induced intestinal inflammation and restores the lifespan of *Drosophila*. (**A**–**I**) RT-qPCR measurement of the *Imd* (**A**), *Relish* (**B**), and its target antimicrobial peptide *gene Attacin A* (**C**), *Diptericin* (**D**), JNK regulatory gene *Mmp1* (**E**), and *MtnA* (**F**) JAK-STAT regulatory genes *upd2* (**G**), *upd3* (**H**), and *Stat92E* (**I**) expression in the gut of control and experimental groups. (**J**) Survival assay in female flies of control and experimental groups. Asterisks denote statistically significant differences (* *p* < 0.05, ** *p* < 0.01, **** *p* < 0.0001). *p*-values were calculated by one-way ANOVA followed by Tukey’s post hoc test; Error bars denote mean ± SEM. *n* = 4 independent experiments.

Excessive ROS generation induced by DSS is often associated with intestinal barrier damage and immune inflammation, thus shortening the survival of *Drosophila* [31,32]. To analyze whether *L.f* ZC529 has anti-inflammatory effects, we measured the expression of antimicrobial peptides (AMPs) and inflammatory cytokines in the *Drosophila* gut. The data showed that when stimulated by 5% DSS, the expression of the key intestinal *immune deficiency* (*Imd*) pathway and its downstream regulator *Relish* as well as its target antimicrobial peptides (*Attacin A* and *Diptericin*) were significantly increased (Figure 4A–D). Additionally, the stress-responsive c-Jun *n*-terminal kinases (JNKs) pathway and its regulatory genes *Mmp1* and *MtnA*, as well as inflammatory cytokines *upd2*, *upd3*, and *stat92E*, were also drastically upregulated (Figure 4E–I), indicating the intestinal inflammation phenotype in the *Drosophila* gut. Interestingly, flies pre-colonized with *L.f* ZC529 greatly decreased the expression of AMPs, inflammatory cytokines, and JNK regulatory genes (Figure 4A–I), suggesting that *L.f* ZC529 alleviates DSS-induced intestinal inflammation. Next, we conducted survival assays in these flies and found that 5% DSS treatment significantly shortened the adult lifespan when compared to control (Figure 4J), while intestinal *L.f* ZC529 colonization restored the survival rate of *Drosophila* (Figure 4J).

### 3.5. L.f ZC529 Ameliorates DSS-Induced Oxidative Stress in IPEC-J2 Cells via Activating the Keap1-Nrf2 Pathway

**Figure 5:**
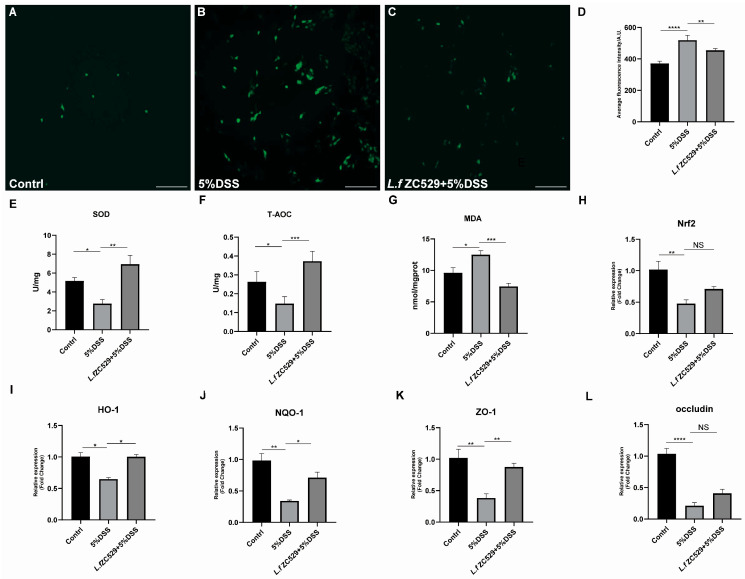
*L.f* ZC529 ameliorates DSS-induced oxidative stress in porcine intestinal epithelial cells via activating the Keap1-Nrf2 pathway. (**A**–**D**) Representative images of antioxidative activity in the IPEC-J2 cells of control (**A**), 5%DSS (**B**), and 5%DSS+*L.f* ZC529 (**C**) cells and their quantification data (**D**). (**E**,**L**) Quantification of the expression of antioxidant enzymes SOD (**E**), T-AOC (**F**), MDA (**G**), Nrf2 (**H**), and its target genes HO-1 (**I**), NQO-1 (**J**), intestinal barrier regulatory genes ZO-1 (**K**), and Occludin (**L**) in the IPEC-J2 cells of control and experimental groups. Asterisks denote statistically significant differences (* *p* < 0.05, ** *p* < 0.01, *** *p* < 0.001, **** *p* < 0.0001). *p*-values were calculated by one-way ANOVA followed by Tukey’s post hoc test; error bars denote mean ± SEM. *n* = 4 independent experiments. Scale bars: 200 µm.

To assess whether *L.f* ZC529 has conserved cytoprotective function, we evaluated its anti-oxidative effects in the porcine intestinal epithelial (IPEC-J2) cell line. As expected, 5% DSS treatment induced a high ROS activity when compared to control (Figure 5A,B). Strikingly, *L.f* ZC529 pre-colonization significantly reduced the intracellular ROS activity (Figure 5C,D). Moreover, we found that the cellular SOD activity and T-AOC level were significantly decreased, while the MDA level was greatly increased in IPEC-J2 cells upon 5%DSS treatment (Figure 5E–G). By contrast, co-cultured IPEC-J2 cells with *L.f* ZC529 significantly increased the activities of SOD and T-AOC but decreased the MDA level. Furthermore, 5%DSS treatment inhibited the expression of Nrf2 and its downstream target genes HO-1 and HQO-1, as shown in Figure 5H–J. The expression of two tight junction proteins, Zonula Occludens-1 (ZO-1) and Occludin, were also significantly decreased upon 5%DSS treatment (Figure 5K,L), suggesting that DSS-induced oxidative stress disrupts normal intestinal barrier function. Strikingly, *L.f* ZC529 pre-colonization significantly restored the expression of these genes in IPEC-J2 cells in response to 5%DSS treatment (Figure 5H–L), indicating that *L.f* ZC529 alleviates DSS-induced oxidative stress in IPEC-J2 cells via the activation of the Keap1-Nrf2 pathway.

### 3.6. L.f ZC529 Alleviates DSS-Induced Inflammatory Response in IPEC-J2 Cells via Inhibiting the TLR4-NF-κB Signaling Pathway

**Figure 6:**
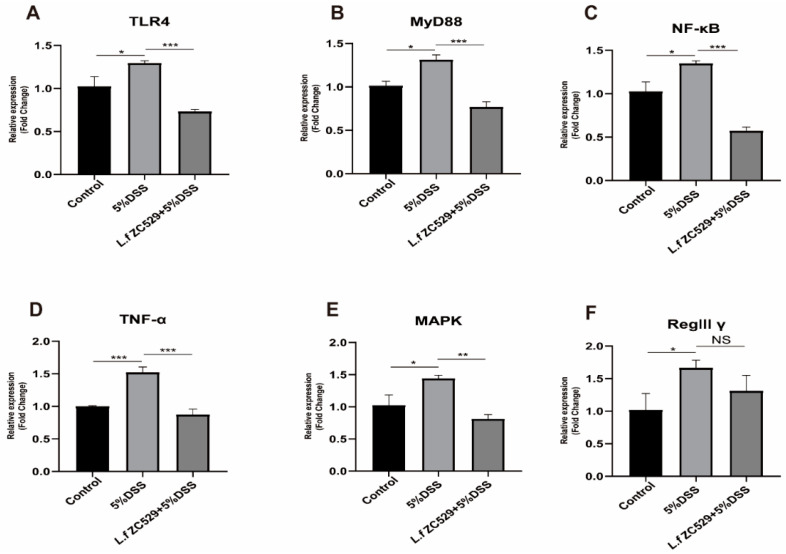
*L.f* ZC529 modulates inflammatory reactions via inhibition of the TLR4-NF-κB pathway. (**A**–**F**) RT-qPCR quantification of the expression of TLR4 (**A**), MyD88 (**B**), NF-κB p65 (RelA) (**C**), TNF-α (**D**), MAPK8 (JNK1) (**E**), and Reg III-γ (**F**) in the IPEC-J2 cells of control and experimental groups. Asterisks denote statistically significant differences (* *p* < 0.05, ** *p* < 0.01, *** *p* < 0.001). *p*-values were calculated by one-way ANOVA followed by Tukey’s post hoc test; error bars denote mean ± SEM. *n* = 4 independent experiments.

We further explored the expression of core genes related to the NF-κB pathway and found that the upstream regulators TLR4, MyD88, NF-κB, and their downstream target genes TNF-α were significantly upregulated after 5% DSS treatment in IPEC-J2 cells (Figure 6A–D). Additionally, the level of MAPK pathway and its regulatory antimicrobial peptide Reg IIIγ expression were also significantly increased in IPEC-J2 cells upon DSS treatment (Figure 6E,F). Interestingly, *L.f* ZC529 pre-colonization decreased the activity of the TLR4-NF-κB signaling pathway and the expression of its target immune and inflammatory effective genes in IPEC-J2 cells in response to DSS treatment (Figure 6A–F), implying its anti-inflammatory effect. These results indicate that *L.f* ZC529 alleviates DSS-induced intestinal immune and inflammatory reactions in IPEC-J2 cells by inhibiting the TLR4-NF-κB signaling pathway.

## 4. Discussion

The modern intensive pig farming generates both acute and chronic stresses that generate excess free radicals, which in turn disrupt the redox balance, leading to oxidative damage and intestinal diseases [33,34]. A variety of oxidative stresses induced by weaning as well as dietary and environmental challenges have been reported to increase diarrhea incidence in piglets, causing vast economic loss [35,36]. It is of great importance to develop new antioxidants to protect the porcine intestine from excessive cellular stress-induced deleterious damage.

The Gram-positive bacteria strain *L.f* ZC529 has been previously isolated from the colon of the DSE pig, one of the Chinese indigenous pig breeds with high stress resistance. By performing phylogenetic analysis, it has been identified as one of the *Limosilactobacillus fermentum* species and exhibits dominant in vitro antioxidant activity [11,12]. Therefore, *L.f* ZC529 may act as probiotic bacteria like *Lactobacillus* to exhibit its in vivo cytoprotective roles upon environmental stresses. In this study, we analyzed the anti-oxidative and anti-inflammatory activities of *L.f* ZC529 in both *Drosophila* and JPEC-J2 cell lines and found that *L.f* ZC529 facilitates its intestinal protective function through the activation of the Keap1-Nrf2 pathway and inhibition of the NF-κB pathway. Specifically, *L.f* ZC529 promoted the expression of antioxidant enzymes to reduce the intestinal damage caused by oxidative stress. In addition, *L.f* ZC529 also protected intestinal epithelial cells from DSS-induced injury by alleviating inflammatory responses. Our findings not only broaden our understanding of the intestinal protective mechanism of probiotic bacteria colonized in indigenous pig breeds but also provide a strategy for the development of *L.f* ZC529 as new antioxidants/probiotics to prevent excessive oxidative stress-induced severe diseases, including diarrhea in pig production.

Oxidative stress often leads to the production of reactive oxygen species (ROS), which oxidizes the cysteine residues on Keap1, leading to its conformational change and release of the Nrf2 from the Keap1-Nrf2 complex. Then, the Nrf2 translocates into the nucleus to interact with small musculoaponeurotic fibrosarcoma proteins and binds to the antioxidant response elements located on antioxidant and detoxification-related genes to promote their transcription to enhance cellular defenses against oxidative stress [37,38,39]. It is known that the activation of the Keap1-Nrf2 pathway initiates antioxidant activity and maintains redox homeostasis in the cell [40]. In our study, we demonstrated that DSS treatment induced a high level of ROS to disrupt the integrity and excretory function of the *Drosophila* gut. In agreement with the anti-oxidative stress activity of the Keap1-Nrf2 pathway, we found that the inclusion of *L.f* ZC529 attenuated DSS-induced intestinal damages in both *Drosophila* and IPEC-J2 cell lines by promoting the expression of antioxidant enzymes CAT and SOD and their downstream antioxidant genes *gstd1*, HO-1, and NQO-1 through the Keap1-Nrf2 pathway. It is noteworthy that, although *L.f* ZC529 markedly mitigated DSS-elicited oxidative stress, a modest elevation of basal ROS levels and a slight reduction in ‘Smurf’-negative flies were observed in the probiotic-only group (Figure 2B and Figure 3B). This phenomenon may reflect transient metabolic activation during early colonization, as reported for other *Lactobacillus* strains [1,2,10]. Nonetheless, these changes did not translate into reduced lifespan or overt epithelial damage, indicating that the benefits of sustained Keap1–Nrf2 activation outweigh the mild oxidative stimulus. Future work should clarify whether probiotic dosage or temporal dynamics fine-tune host redox homeostasis.

Additionally, the TLR4-NF-κB pathway acts as an important immune and inflammatory signaling pathway involved in cellular responses to pathogens or injury signals [41]. The NF-κB pathway plays a central role in modulating the inflammatory response upon different chemical stimuli treatment [42]. TLR4 is a pattern recognition receptor located on cell membranes that primarily recognizes endotoxins and other pathogen/damage-associated molecular patterns [43]. Upon TLR4 activation, the adaptor protein MyD88 is recruited to the receptor complex, triggering downstream activation of the NF-κB transcription factor (mainly the p65/RelA–p50 heterodimer), which initiates the transcription of pro-inflammatory genes such as TNF-α, IL-1β, and IL-6; excessive activation of the TLR4–NF-κB pathway ultimately results in inflammation and cellular damage [44,45,46]. Furthermore, the classical stress-responsive JNK signaling pathway has been shown to be activated by various oxidative stresses, and its activation stimulates the downstream JAK/STAT signaling pathway to induce inflammatory responses [47]. Our results found that DSS stimulation elevated the NF-κB and JNK/JAK/STAT92E activities and the expression of antimicrobial peptides, inflammatory cytokines, and stress-responsive genes in the *Drosophila* gut. However, intestinal *L.f* ZC529 colonization was able to attenuate the DSS-induced inflammatory responses and intestinal barrier dysfunction by inhibiting the NF-κB and JNK/JAK/STAT92E signaling pathways. Meanwhile, the expression of two key tight junction proteins, ZO-1 and Occludin, was also restored after *L.f* ZC529 supplementation in response to DSS treatment in IPEC-J2 cells. Taken together, the underlying mechanisms of *L.f* ZC529 in protecting intestinal epithelium from DSS-induced injury likely coordinate both anti-oxidative and anti-inflammatory signaling pathways. Of course, we cannot exclude another possibility that the intestinal cytoprotective effect of *L.f* ZC529 on the intestine is achieved by modulation of the composition and diversity of gut microbiota; this possibility awaits further investigation.

Probiotic bacteria like *Lactobacillus* and its generated postbiotics are involved in modulating intestinal immune responses and also act as antioxidants to attenuate intestinal oxidative stresses, thus maintaining intestinal homeostasis. It has been shown that the new fatty acid metabolite of linoleic acid produced by *Lactobacillus plantarum* in the gut protects human hepatocellular carcinoma cell line G2(HepG2) cells from hydrogen peroxide-induced cytotoxicity, by enhancing the Keap1-Nrf2 activity and its downstream target HO-1, GCLM, and NQO1 gene expression [48]. In addition, *Lactobacillus plantarum* Y44 exerts anti-oxidative effects by quenching oxygen radicals and activating the Keap1-Nrf2 signaling pathway in Caco-2 cells [49]. Similarly, Zheng et al. [50] isolated *Lactobacillus rhamnosus* CY12 (LCY12) from cow’s milk and found that LCY12 enhanced the expression of tight junction proteins and inhibited the TLR4-NF-κB signaling pathway to attenuate the DSS-induced colitis in mice. Our study revealed the profound anti-oxidative effects of *L.f* ZC529 in the intestinal epithelium cells and implied its potential application as probiotic bacteria to maintain gut health. Thus, it is likely that these probiotic bacteria isolated from animals manifest the conserved intestinal cytoprotective function. Whether *L.f* ZC529 generates any secondary metabolites or acts together with other probiotic bacteria to fulfill its anti-oxidative activity needs further examination.

Although the clear antioxidative and anti-inflammatory effects of *L.f* ZC529 have been validated both in *Drosophila* and IPEC-J2 cell models, our study still has several limitations. Firstly, we did not address whether *L.f* ZC529 colonization alters the composition and diversity of gut microbiota in vivo. The gut microbiota has been shown to play a critical role in maintaining gut homeostasis by interacting with the host immune systems. It is possible that *L.f* ZC529 may compete or cooperate with other commensal gut flora to modulate gut immune signaling to prevent gut epithelium from DSS-induced injury. This hypothesis needs further experimental validation. Secondly, the in vivo activity of *L.f* ZC529 in higher organisms is still not fully investigated. IPEC-J2 cells are isolated from the jejunum of a neonatal piglet, but this cell line cannot mimic the 3D structure of the porcine gut. Therefore, a pig model combined with metagenomic and metabolomic analyses can be employed in the future to investigate how *L.f* ZC529 facilitates its intestinal protective function by interacting with gut microorganisms and regulating immune and metabolic reactions. This future research direction will expand the mechanistic understanding of the antioxidative and anti-immune effects of *L.f* ZC529 in vivo and also enhance its translational application in animal production.

## 5. Conclusions

In conclusion, by using two different DSS-induced oxidative models, we unraveled the mechanisms by which the *L.f* ZC529 modulated intestinal epithelium cytoprotection through the activation of the Keap1-Nrf2 signaling pathway and inhibition of the NF-κB signaling pathways. The application of the anti-oxidative and anti-inflammatory activities of *L.f* ZC529 as a new antioxidant or probiotic to improve gut health may bring large benefits for pig production.

## Figures and Tables

**Table 1:** List of qPCR primers used in *Drosophila*.

Drosophila	Primers Sequence (5′–3′)	Accession No.	Length (bp)	References
***Rp49***	F:ATCGGTTACGGATCGAACAAGC R: GTAAACGCGGTTCTGCATGAGC	Y13939.1	148	[24,25,26]
***CAT***	F: TTCCTGTGGGCAAAATGGTG R: ATCTTCACCTTGTACGGGCA	NM_080483.3	134	
***SOD***	F: CAAGGGCACGGTTTTCTTC R: CCTCACCGGAGACCTTCAC	NM_057387.5	120	
***gstd1***	F: CATCGCGAGTTTCACAACAG R: GTTGAGCAGCTTCTTGTTCAG	NM_001038953.2	141	
***keap1***	F: CAAGGAGTCGGAGATGTCG R: GTAGAGGATGCGTGACATGG	DQ372684.1	150	
***Attacin A***	F: GCATCCTAATCGTGGCCCT R: AGCGGGATTGGAGGTTAAGG	NM_079021.5	132	
***Diptericin***	F: CTCAATCTTCAGGGAGGCGG R: AGGTGCTTCCCACTTTCCAG	NM_057460.4	125	
***Imd***	F: GCTCCGTCTACAACTTCAACC R: CCACAATGCTGACCGTTTTG	NM_133166.4	140	
***Relish***	F: GGTCCAGCTGCTGAAGAATG R: ACGGAATCCTCGTCCTTTGT	NM_057746.4	128	
***Mmp1***	F: AGGACTCCAAGGTAGACACAC R: TTGCCGTTCTTGTAGGTGAACGC	NM_001259570.2	138	
***MtnA***	F: TGCAAATGCGCCAGCCAG R: TCGGAGCAGCCGCAGG	NM_079575.2	142	
***upd2***	F: CGGAACATCACGATGAGCGAAT R: TCGGCAGGAACTTGTACTCG	NM_001370039.1	129	
***upd3***	F: ATCCCACCAATCCCCTGAAG R: AGATTGCAGGTGTTCTCCCA	NM_001103544.2	130	
***STAT92E***	F: AGTTCTACTCAAAGCGTCAAGATCC R: CAGTTGCATGCTTTCCTGAGC	NM_001275833.1	146	

**Table 2:** List of qPCR primers used in IPEC-J2.

IPEC-J2	Primers Sequence (5′–3′)	Accession No.	Length (bp)	References
***β-actin***	F: CCAGGTCATCACCATCGGCAAC R: CAGCACCGTGTTGGCGTAGAG	DQ845171.1	143	[27,28]
***SOD2***	F: GGCCTACGTGAACAACCTGA R: TGATTGATGTGGCCTCCACC	NM_214127.2	135	
***CAT***	F: AGCCTACGTCCTGAGTCTCTGC R: TCCATATCCGTTCATGTGCCTGTG	NM_214301.2	126	
***Nrf2***	F: AGCCTACGTCCTGAGTCTCTGC R: TCCATATCCGTTCATGTGCCTGTG	MH101365.1	119	
***Keap1***	F: CGTGGAGACAGAAACGTGGA R: CAATCTGCTTCCGACAGGGT	NM_001114671.1	131	
***HO-1***	F: TGATGGCGTCCTTGTACCAC R: GACCGGGTTCTCCTTGTTGT	NM_001004027.1	115	
***NQO-1***	F: CATGGCGGTCAGAAAAGCAC R: ATGGCATACAGGTCCGACAC	NM_001159613.1	129	
***ZO-1***	F: CAGAGACCAAGAGCCGTCC R: TGCTTCAAGACATGGTTGGC	AJ318101.1	140	
***Occludin***	F: TCAGGTGCACCCTCCAGATT R: AGGAGGTGGACTTTCAAGAGG	NM_001163647.2	137	
***TLR4***	F: GACAGCAATAGCTTCTCCAGC R: GGTTTGTCTCAACGGCAACC	NM_001113039.2	132	
***MyD88***	F: GTGCCGTCGGATGGTAGTG R: TCTGGAAGTCACATTCCTTGCTT	EU056737.1	145	
***MAPK8 (JNK1)***	F: CAGCCGATTCGGAGCACAACA R: GGTGGTGGAGCTTCAGCTTCAG	NM_001143717.1	132	
***NF-κB p65 (RelA)***	F: AAGCAGAGCCGCACAGCATTC R: CCAGACCAACAACAACCCCTTCC	EU399817.1	138	
***TNF-α***	F: AACCTCAGATAAGCCCGTCG R: ACCACCAGCTGGTTGTCTTT	JF831365.1	141	
***Reg*** ***Ⅲ*** ***γ***	F: GGCTTGGAACCAAATGCTGG R: TAGCCAGGGTATGAGCTGGT	NM_001144847.1	133	

## Data Availability

This study did not generate any unique code.
